# Age-related changes of dopamine D1 and D2 receptors expression in parvalbumin-positive cells of the orbitofrontal and prelimbic cortices of mice

**DOI:** 10.3389/fnins.2024.1364067

**Published:** 2024-06-06

**Authors:** Jihui Dong, Xiaoyan Wei, Ziran Huang, Jing Tian, Wen Zhang

**Affiliations:** ^1^Department of Neurobiology, School of Basic Medical Sciences, National Institute on Drug Dependence, Peking University, Beijing, China; ^2^Beijing Key Laboratory of Drug Dependence Research, Peking University, Beijing, China

**Keywords:** dopamine receptor, D1, D2, orbitofrontal cortex, prefrontal cortex, development, age

## Abstract

Dopamine (DA) plays a pivotal role in reward processing, cognitive functions, and emotional regulation. The prefrontal cortex (PFC) is a critical brain region for these processes. Parvalbumin-positive (PV+) neurons are one of the major classes of inhibitory GABAergic neurons in the cortex, they modulate the activity of neighboring neurons, influencing various brain functions. While DA receptor expression exhibits age-related changes, the age-related changes of these receptors in PV+ neurons, especially in the PFC, remain unclear. To address this, we investigated the expression of DA D1 (D1R) and D2 (D2R) receptors in PV+ neurons within the orbitofrontal (OFC) and prelimbic (PrL) cortices at different postnatal ages (P28, P42, P56, and P365). We found that the expression of D1R and D2R in PV+ neurons showed both age- and region-related changes. PV+ neurons in the OFC expressed a higher abundance of D1 than those in the PrL, and those neurons in the OFC also showed higher co-expression of D1R and D2R than those in the PrL. In the OFC and PrL, D1R in PV+ neurons increased from P28 and reached a plateau at P42, then receded to express at P365. Meanwhile, D2R did not show significant age-related changes between the two regions except at P56. These results showed dopamine receptors in the prefrontal cortex exhibit age- and region-specific changes, which may contribute to the difference of these brain regions in reward-related brain functions.

## 1 Introduction

The prefrontal cortex (PFC) plays a central role in various cognitive processes, including working memory, decision-making, attention, emotion, and memory (Fuster and Alexander, 1971; Dias et al., 1996; Ochsner and Gross, 2005; Gold and Shadlen, 2007; Goldman-Rakic, 2011). It is vulnerable to multiple neurological and psychological diseases, such as depression, schizophrenia, and addiction (Lewis et al., 2005; Belmaker and Agam, 2008; Koob and Volkow, 2010). The PFC of rodents can be subdivided into four primary subregions, extending from dorsal to ventral: cingulate, prelimbic (PrL), infralimbic (IL), and orbitofrontal (OFC) cortices (Brodmann, 1909; Rose and Woolsey, 1948a,b; Caviness, 1975; Uylings and van Eden, 1990; Zilles, 2012). While both the PrL and OFC are involved in decision-making and emotional regulation (Zeeb et al., 2015; Mızrak et al., 2021; Hernandez et al., 2022), they exhibit distinct functions. The PrL is more associated with executive functions (Baker and Ragozzino, 2014; Broschard et al., 2021) and emotional regulation (Mears et al., 2009), whereas the OFC plays a particular role in the evaluation of rewards and punishments, guiding appropriate behavioral responses (Lichtenberg et al., 2021; Frontera et al., 2023). Dysfunction in the PrL has been implicated in disorders such as anxiety (Luo et al., 2023). In contrast, damage to the OFC can lead to changes in social behavior and decision-making (Bolla et al., 2003; du Plessis et al., 2018). The PFC undergoes age-related changes both in cognitive functions and brain substrates (West, 1996; Chao and Knight, 1997). The PFC shows structural changes associated with increased functional connections during development (Park et al., 2021; Sydnor et al., 2021). Meanwhile, the working memory, which the PFC encodes task-relevant information with, also improves until puberty in humans and primates (Bunge et al., 2002; Gathercole et al., 2004; Zhou et al., 2014). On the other hand, in adults, both working memory and functional connections of prefrontal brain regions show disproportionately strong age-related declines (Salthouse et al., 2003; Hedden and Gabrieli, 2004; Zhou et al., 2014).

In the cortex, the orchestrated activity of inhibition and excitation is critical for brain functions (Zhang et al., 2014, 2017). Recent studies have shown that disinhibition is implicated in several psychological disorders, such as schizophrenia (Kokkinou et al., 2021), depression (Kantrowitz et al., 2021) and attention-deficit/hyperactivity disorder (Niedermeyer, 2001). Cortical inhibition is mainly mediated by GABAergic interneurons, the major types of which are parvalbumin-, somatostatin-, and vasoactive intestinal peptide-positive (PV+, SST+, and VIP+, respectively) interneurons. These neurons show diverse morphology, axon targeting location, and functional differences, which are manifested by their roles in circuit and brain functions, such as oscillation. Of these neurons, PV+ neurons show a consistent inhibitory effect on local excitatory pyramidal neurons (Zhang et al., 2016). Such a property makes PV+ neurons critical for cortical inhibition and functions, and studies have shown that changes in PV+ neuron activity or the synaptic strength of PV+ to layer 5 excitatory neurons changed cortical output and brain functions (Lee et al., 2014; Sempere-Ferràndez et al., 2019).

Both the PrL and OFC are crucial components of a vital brain circuit known as the reward pathway (Zhang, 2020). In the reward pathway, dopamine serves as a pivotal neurotransmitter. Dopamine is suggested as a reward signal in the brain for activities that controls animal actions, decisions, and choices, and acting as a reward signal that signals the discrepancy between the actual reward and its prediction (Lee et al., 2021; Farrell et al., 2022; Seitz et al., 2022). Dopamine receptors are G protein-coupled receptors, classified into two main types: D1-like receptors, including D1 and D5 subunits, and D2-like receptors, comprising D2–D4 subunits. These two types of receptors engage in distinct downstream signaling cascades within neurons. D1-like and D2-like receptors have opposing effects on adenylyl cyclase activity and cAMP concentration, as well as on phosphorylation of Dopamine- and cAMP-regulated neuronal phosphoprotein (DARPP-32) (Hemmings and Greengard, 1986; Nishi et al., 1997; Greengard et al., 1999). By phosphorylation (facilitated by D1-like and inhibited by D2-like receptors), DARPP-32 inhibits the protein phosphatase PP-1, which modulates the activity of various voltage-gated and synaptic ion channels. For example, activation of D1-like receptors increased the intrinsic excitability of neurons, including PV+ neurons (Potts and Bekkers, 2022; Plateau et al., 2023). Interestingly, in adolescent rats the modulation is exclusively D1-mediated, while in older animals a D2-mediated modulation is synergistic with the D1-mediated effect (Tseng and O’Donnell, 2007). For synaptic transmissions, dopamine enhances the amplitude of NMDA synaptic currents via D1 receptors and reduces via D2 receptors in the PFC (Chen and Yang, 2002; Banks et al., 2015); it also enhances GABAergic currents via D1-like receptors and reduces them via D2-like receptors in the striatum and PFC (Seamans et al., 2001; Ji et al., 2009).

Given the modulatory effects of D1-like and D2-like dopamine receptors on neuronal activity and the crucial role of PV+ interneurons in the circuit activities of cortical regions, it is essential to delineate the age-related changes of dopamine receptor expression profiles in PV+ neurons within the PrL and OFC to comprehend their contributions to brain functions. To address this question, we examined the expression patterns of dopamine D1 and D2 receptors in PV+ neurons in the OFC and PrL and compared age-related expression changes of these receptors in mice.

## 2 Materials and methods

### 2.1 Animals

Male C57BL/6J mice (RRID: IMSR_JAX:000664) were used. Mice were maintained on a 12 h light/dark cycle with food and water *ad libitum*. All experiments were performed in the dark cycle.

All procedures are in accordance with the National Institutes of Health *Guide for the Care and Use of Laboratory Animals* and have been approved by Peking University Animal Care and Use Committee.

### 2.2 Immunostaining

For immunostaining, mice were anesthetized with isoflurane, then perfused with phosphate-buffer saline (PBS, pH 7.4) followed with 4% paraformaldehyde (PFA) in PBS. Brains were dissected and post-fixed with 4% PFA in PBS overnight at 4°C, and 25 μm coronal sections were prepared with a vibratome. We chose sections in the range of stereotaxic position Bregma +2.68 to 1.7 mm, according to an atlas of the adult mice (Paxinos et al., 2001). Of mice for whom these positions were not suitable, we selected sections based on the existence of the rhinal fissure, lateral ventricle, or the forceps minor of the corpus callosum. Immunostaining followed the standard protocols for free-floating sections. In brief, free-floating sections were incubated in blocking solution containing 4% normal donkey serum, 1% bovine serum albumin (BSA), and 0.3% Triton X-100 in PBS for 2 h at 23–25°C. Sections were then treated with primary antibodies in blocking solution for 24–48 h at 4°C, followed with secondary antibodies in blocking solution at 23–25°C for 2 h with slow shaking.

Primary antibodies used were Goat Anti-Parvalbumin (1:2000, Swant, Cat# PVG-213, RRID: AB_2650496), Rat Anti-Dopamine D1 Receptor (1:200, Sigma, Cat# D2944, RRID: AB_1840787), Rabbit Anti-Dopamine D2 Receptor (1250, Merck, Cat# AB5084P, RRID: AB_2094980).

Secondary antibodies used were Alexa Fluor 546 Anti-Goat (1:300, Thermo Fisher Scientific, Cat# A11056, RRID: AB_2534103), Alexa Fluor 488 Anti-Rat (1:300, Abcam, Cat# ab150153, RRID: AB_2737355), Alexa Fluor Plus 647 Anti-Rabbit (1:300, Thermo Fisher Scientific, Cat# A32795, RRID: AB_2762835).

### 2.3 Antibody characterization

The parvalbumin antiserum (Swant, Cat# PVG-213), made against rat muscle parvalbumin, recognized the monomeric (12 kD) and dimer (24 kD) bands on Western blot of mice brain. Staining with this antibody was eliminated in parvalbumin knock-out mice (manufacturer’s datasheet).

The dopamine D1 receptor antibody (Sigma, Cat# D2944) was derived from the rat hybridoma 1-1-F11 S.E6 produced by the fusion of mouse myeloma cells and splenocytes from rat immunized with recombinant fusion protein containing the C-terminal 97 amino acid of human D1 dopamine receptor.

The dopamine D2 receptor antibody (Merck, Cat# AB5084P) was raised against a 28 amino acid peptide sequence from the human D2 receptor within the cytoplasmic loop #3, and recognized a band of 50 kD on Western blots of mouse brain (manufacturer’s datasheet).

The dopamine D1 and D2 receptor antibodies were specific for D1 and D2 in mouse as evaluated by Western blotting and immunohistochemistry, and confirmed by the immunoprecipitation with mass spectrometry (Stojanovic et al., 2017).

### 2.4 Imaging

We acquired fluorescent images with a confocal microscope (Leica TCS-SP8 STED) using a 63× objective (NA 1.4) and a 16× objective (NA 0.5). The analysis was performed as previously described (Zhang et al., 2016). Briefly, a maximal projection of a 7 μm thick stack was analyzed with ImageJ (v1.53t, RRID:SCR_003070) based FIJI (RRID:SCR_002285) (Whitesell et al., 2021). The punta of D1 and D2 dopamine receptors of a minimum 2 pixels on parvalbumin-positive cell soma were analyzed with particle analysis of FIJI. The soma areas of PV+ cell, D1, and D2 dopamine receptors puncta size more than 5 times of the standard deviation were excluded from further analysis.

### 2.5 Statistical analysis

All statistical analyses and data plotting were performed with R (v4.2.2, RRID: SCR_001905), and the non-base attached packages for R were ggpubr (v0.6.0, RRID:SCR_021139), rstatix (v0.7.2, RRID:SCR_021240), tidyverse (v2.0.0, RRID:SCR_019186), and emmeans (v1.8.5, RRID:SCR_018734). For boxplots, whiskers denoted 1.5 * IQR from the hinges, which corresponded to the first and third quartiles of distribution. For multiple groups, one-way or two-way ANOVAs with *post hoc* Tukey’s test were used based on experiment design. *n*, sample number of cells; *N*, sample number of mice. *p* < 0.05 is considered statistically significant.

## 3 Results

### 3.1 The age-related changes of parvalbumin-positive neurons in the orbitofrontal and prelimbic cortices

**Figure 1:**
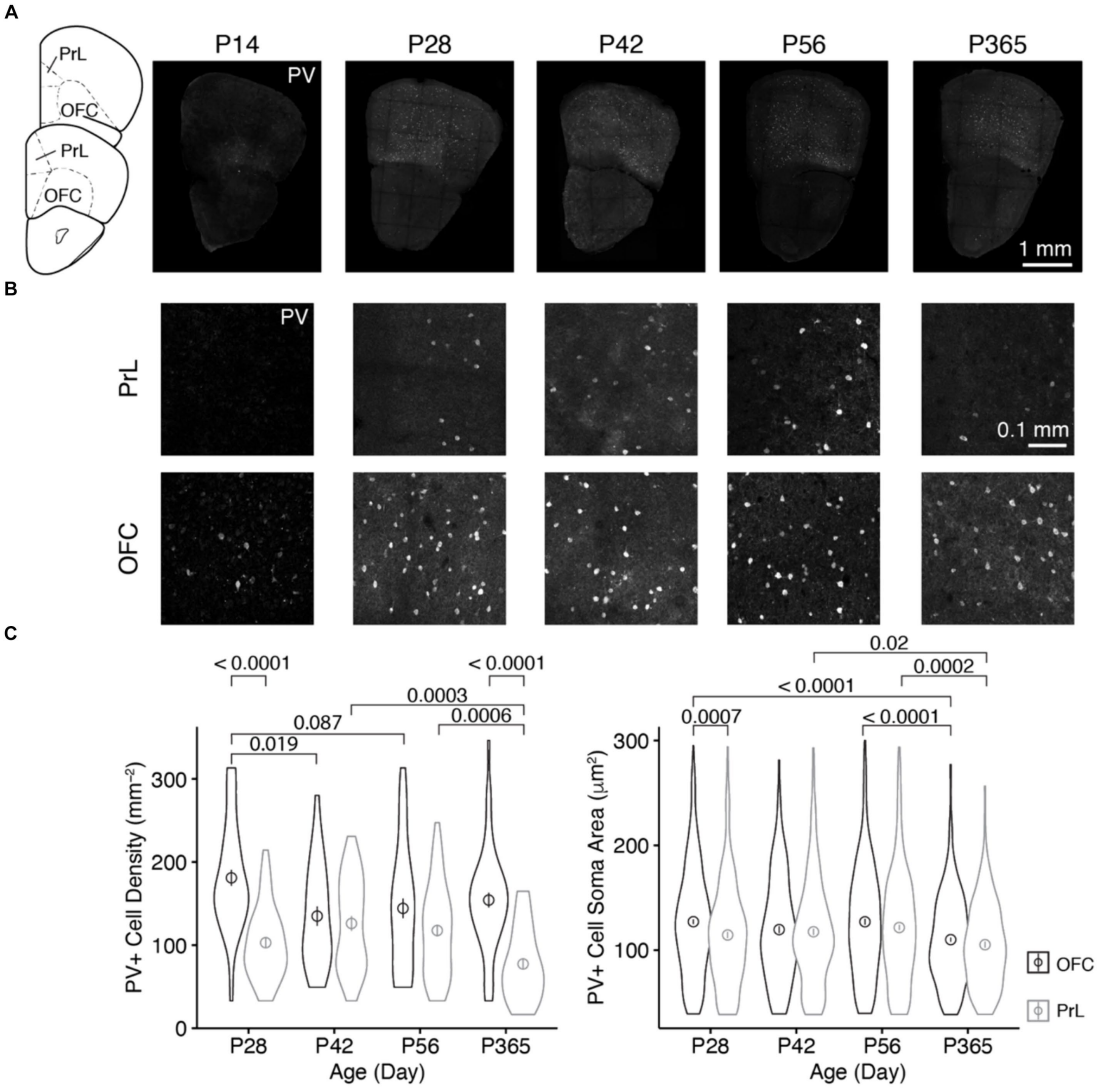
The distribution of PV+ neurons in the OFC and PrL at different ages of mice. **(A)** Brain sections containing the OFC and PrL with PV+ neurons immunolabeled with anti-parvalbumin at different ages. Inset at left, atlas indicating locations of the brain sections. Scale bar, 1 mm. **(B)** Blow-up of the OFC and PrL regions at different ages. Scale bar, 0.1 mm. **(C)** Comparisons of the cell density and soma area of PV+ neurons in the OFC and PrL regions (Left, PV+ cell density: Two-way ANOVA, *F*_1, 332_ (region) = 40.4, *p* = 6.84 × 10^−10^, *F*_1, 332_ (age) = 4.5, *p* = 0.004, *F*_3, 332_ (region: age) = 7.2, *p* = 0.0001; *post hoc* Tukey’s test: P28 of OFC vs. P28 of PrL, *p* = 3.95 × 10^−9^; P42 of OFC vs. P42 of PrL, *p* = 0.55; P56 of OFC vs. P56 of PrL, *p* = 0.06; P365 of OFC vs. P365 of PrL, *p* = 1.75 × 10^−9^. Right, PV+ cell soma area: Two-way ANOVA, *F*_1, 2700_ (region) = 12.0, *p* = 5.52 × 10^−4^, *F*_1, 2700_ (group) = 11.8, *p* = 1.14 × 10^−7^, *F*_3, 2700_ (region: group) = 1.3, *p* = 0.26; *post hoc* Tukey’s test: P28 of OFC vs. P28 of PrL, *p* = 7.36 × 10^−4^; P42 of OFC vs. P42 of PrL, *p* = 0.65; P56 of OFC vs. P56 of PrL, *p* = 0.15; P365 of OFC vs. P365 of PrL, *p* = 0.18. P28 of OFC: cell number, *n* = 528; section, *n* = 44; P28 of PrL: cell number, *n* = 279; section, *n* = 45; P42 of OFC: cell number, *n* = 220; section, *n* = 27; P42 of PrL: cell number, *n* = 281; section, *n* = 37; P56 of OFC: cell number, *n* = 343; section, *n* = 40; P56 of PrL: cell number, *n* = 426; section, *n* = 60; P365 of OFC: cell number, *n* = 449; section, *n* = 48; P365 of PrL: cell number, *n* = 182; section, *n* = 39; *N* = 3 mice/group). Circles and bars in violin plots denote the mean ± sem.

We examined the age-related expressions of dopamine D1 and D2 receptors (D1R and D2R, respectively) in parvalbumin-positive (PV+) neurons in the orbitofrontal (OFC) and prelimbic (PrL) regions of the prefrontal cortex (PFC). During development, parvalbumin began to express in the PFC around 2 weeks old, when the expression of Potassium-chloride transporter member 5 (KCC2) reached to the level of maturation in neurons, which makes the GABAergic transmission by these interneurons inhibitory (Virtanen et al., 2021). Rodents showed enhanced inhibitory transmission and reduced excitation in the cortex around P28, and such changes were reversed by the end of the post-natal sixth week (Zhang et al., 2018). At the post-natal eighth week, mice entered adulthood and the cognitive functions of mice involving the prefrontal cortex reached full maturity (Popplau et al., 2024). Young adult mice (3–8 months old) showed similar cognitive functions, but at middle age (11–14 months old), mice began to show functional alterations of the prefrontal circuit underlying executive functions (Chong et al., 2023). Accordingly, we first examined parvalbumin expression in these two brain regions on post-natal days 14, 28, 42, 56, and 365 (P14, P28, P42, P56, and P365, respectively; Figure 1).

We found that at P14, parvalbumin was barely expressed in the PrL, while the OFC showed strong parvalbumin expression. The cell density at P14 was lower than that at other ages (Figures 1A,B). Thus, in the following analyses, we focused at the older ages, namely, P28, P42, P56, and P365. We found that the density of PV+ cells was higher in the OFC than that in the PrL at both P28 and P365, and the soma area of PV+ cells in the OFC was larger than that in the PrL at P28 (Figure 1C). Furthermore, PV+ cell density showed a decrease in the OFC during development, while that change was not observed in the PrL, but cell density at P365 decreased significantly (Figure 1C). These results indicate different age-related changes in PV+ cells in the two regions.

### 3.2 The age-related changes of dopamine D1 and D2 receptor expressions in PV+ neurons in the OFC and PrL

**Figure 2:**
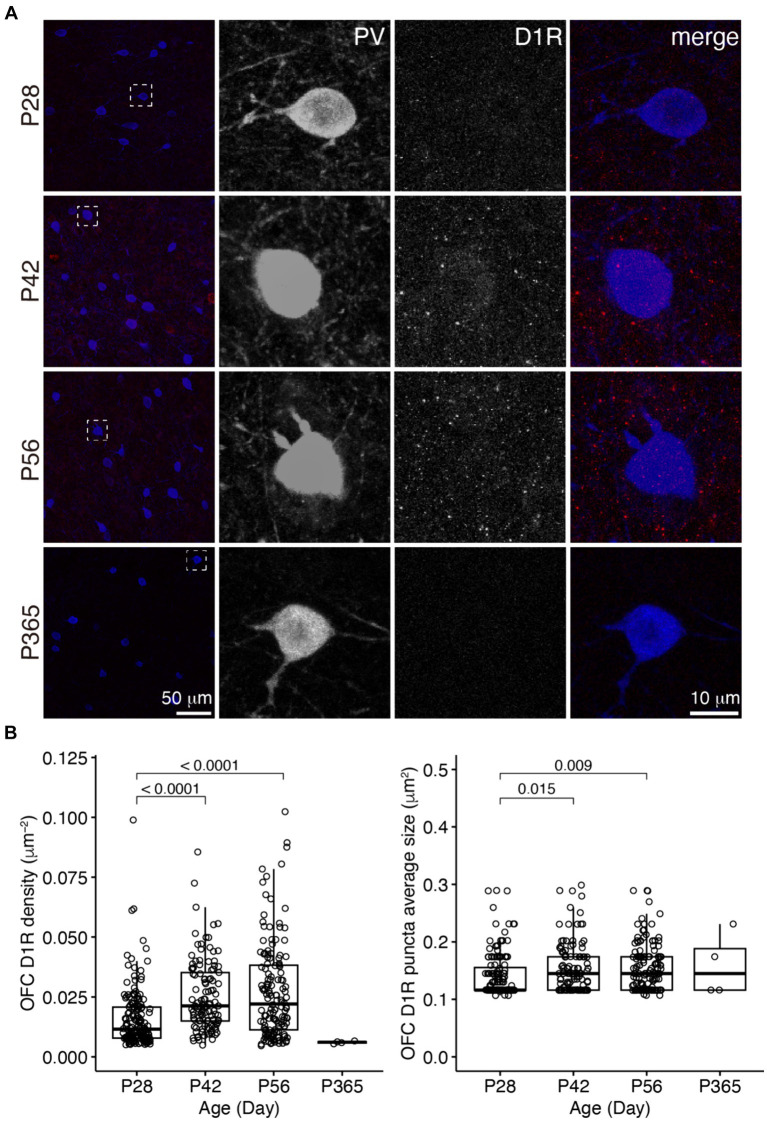
The expression of D1R in PV+ neurons of the OFC. **(A)** The characteristic expression of D1R in PV+ neurons in the OFC at different ages. Three panels on the right show the blow-up of the dashed square in the left panel. Scale bars, 50 and 10 μm. **(B)** The density and puncta average size of D1R expressed by PV+ neurons in the OFC (Left, OFC D1R density: Welch one-way ANOVA, *F*_2, 248.3_ = 24.8, *p* = 1.56 × 10^−10^; *post hoc* Tukey’s test: P28 vs. P42, *p* = 2.27 × 10^−7^; P28 vs. P56, *p* = 7.99 × 10^−8^; P42 vs. P56, *p* = 0.71. Right, OFC D1R puncta average size: Welch one-way ANOVA, *F*_2, 255.1_ = 6.0, *p* = 0.003; *post hoc* Tukey’s test: P28 vs. P42, *p* = 0.02; P28 vs. P56, *p* = 0.009; P42 vs. P56, *p* = 0.99. P28, *n* = 160; P42, *n* = 113; P56, *n* = 142; P365, *n* = 4; *N* = 3 mice/group).

**Figure 3:**
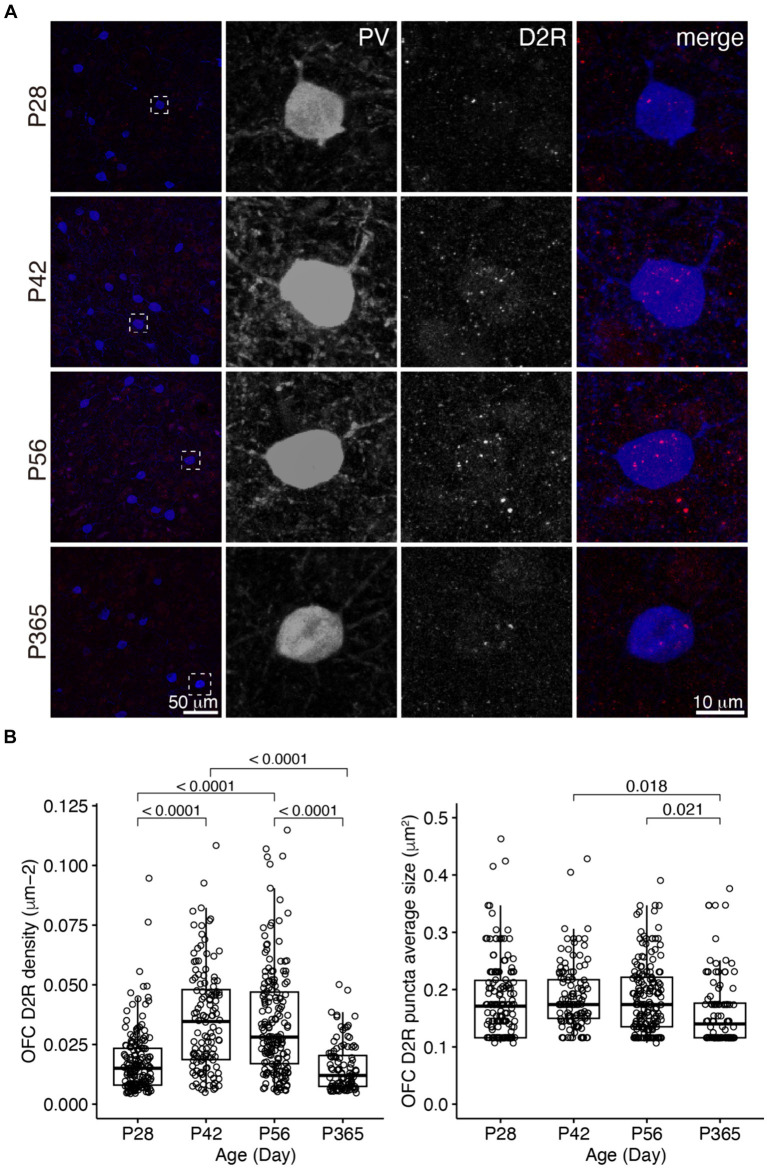
The expression of D2R in PV+ neurons of the OFC. **(A)** The expression of D2R in PV+ neurons. Three panels on the right show the blow-up of the dashed square in the left panel. Scale bars, 50 and 10 μm. **(B)** The density and puncta average size of D2R expressed by PV+ neurons in the OFC (Left, OFC D2R density: Welch ANOVA, *F*_3, 292_ = 51.6, *p* = 8.55 × 10^−27^; *post hoc* Tukey’s test: P28 vs. P42, *p* = 2.35 × 10^−13^; P28 vs. P56, *p* = 6.52 × 10^−13^; P28 vs. P365, *p* = 0.39; P42 vs. P56, *p* = 0.96; P42 vs. P365, *p* = 8.09 × 10^−14^; P56 vs. P365, *p* = 1.54 × 10^−13^. Right, OFC D2R puncta average size: Welch ANOVA, *F*_3, 285_ = 3.5, *p* = 0.02; *post hoc* Tukey’s test: P28 vs. P42, *p* = 0.83; P28 vs. P56, *p* = 0.88; P28vs P365, *p* = 0.15; P42 vs. P56, *p* = 1.00; P42 vs. P365, *p* = 0.02; P56 vs. P365, *p* = 0.02. P28, *n* = 162; P42, *n* = 124; P56, *n* = 171; P365, *n* = 100; *N* = 3 mice/group).

**Figure 4:**
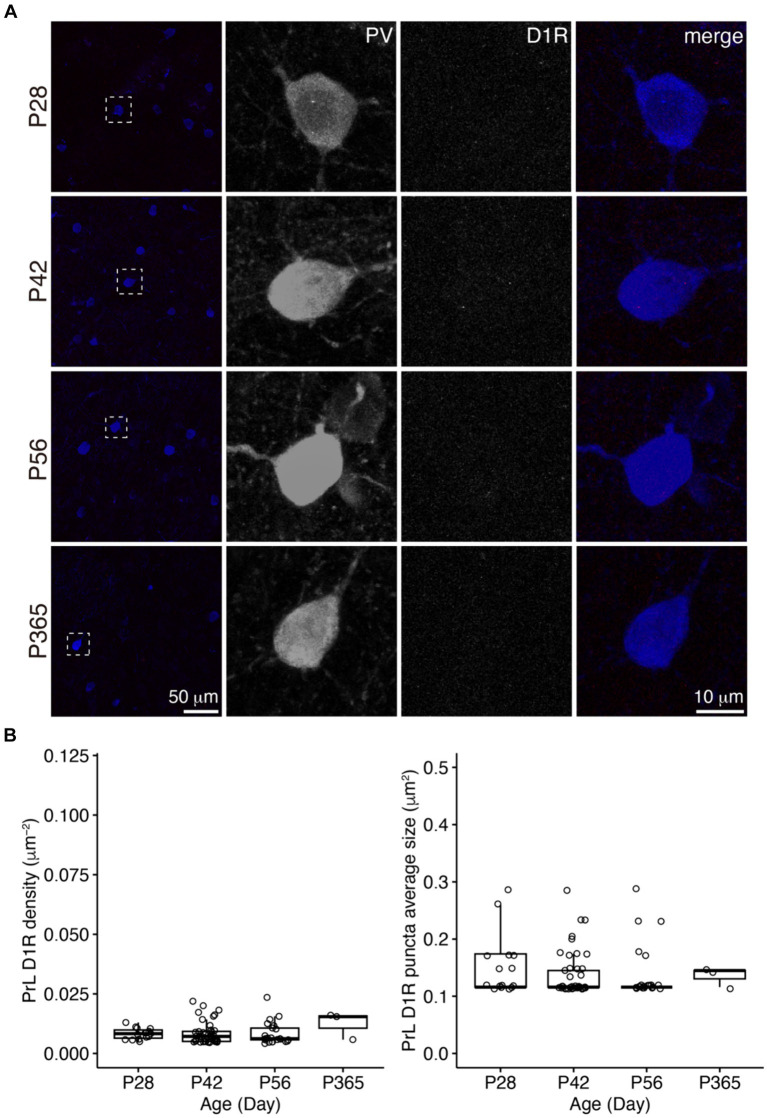
The expression of D1R in PV+ neurons of the PrL. **(A)** The expression of D1R in PV+ neurons. Three panels on the right show the blow-up of the dashed square in the left panel. Scale bars, 50 and 10 μm. **(B)** The density and puncta average size of D1R expressed by PV+ neurons in the PrL (Left, PrL D1R density: Welch one-way ANOVA, *F*_2, 41.7_ = 0.1, *p* = 0.94. Right, PrL D1R puncta average size: Welch one-way ANOVA, *F*_2, 30.7_ = 0.3, *p* = 0.73. P28, *n* = 15; P42, *n* = 40; P56, *n* = 21; P365, *n* = 3; *N* = 3 mice/group).

**Figure 5:**
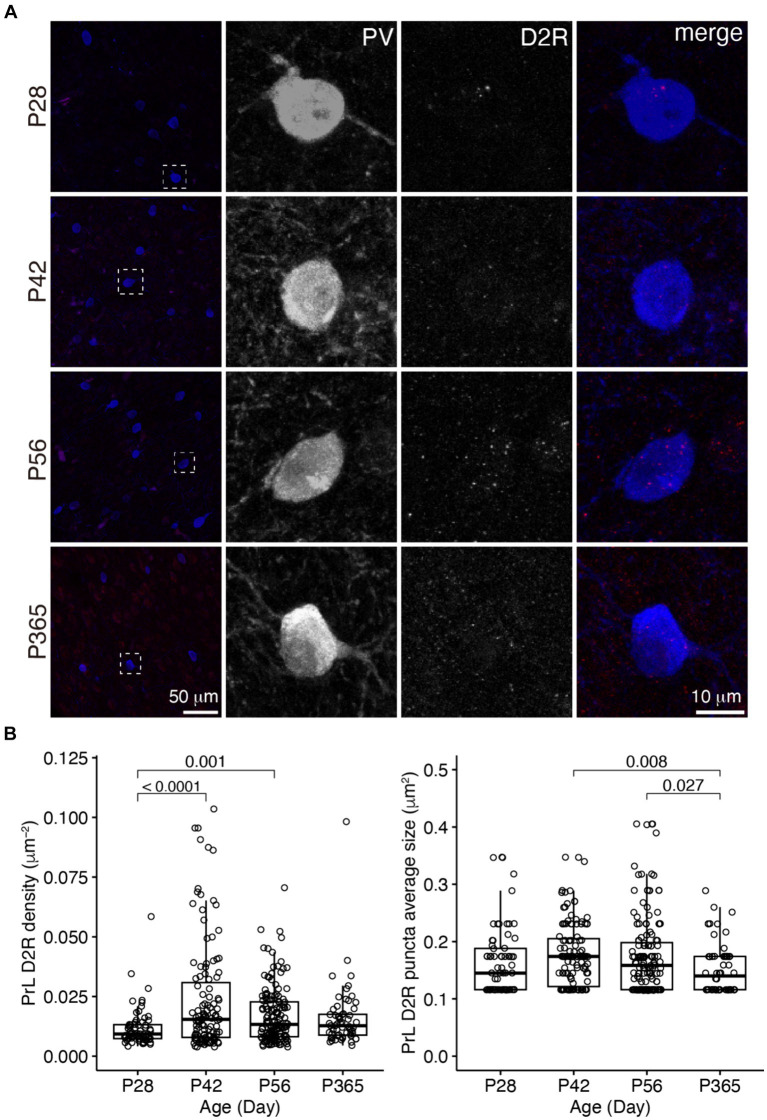
The expression of D2R in PV+ neurons of the PrL. **(A)** The expression of D2R in PV+ neurons. Three panels on the right show the blow-up of the dashed square in the left panel. Scale bar, 50 μm, 10 μm. **(B)** The density and puncta average size of D2R expressed by PV+ neurons in the PrL (Left, PrL D2R density: Welch one-way ANOVA, *F*_3, 177_ = 10.1, *p* = 3.43 × 10^−6^; *post hoc* Tukey’s test: P28 vs. P42, *p* = 8.31 × 10^−6^; P28 vs. P56, *p* = 0.001; P28 vs. P365, *p* = 0.17; P42 vs. P56, *p* = 0.03; P42 vs. P365, *p* = 0.03; P56 vs. P365, *p* = 0.94. Right, PrL D2R puncta average size: Welch one-way ANOVA, *F*_3, 180_ = 4.12, *p* = 0.007; *post hoc* Tukey’s test: P28 vs. P42, *p* = 0.58; P28 vs. P56, *p* = 0.75; P28 vs. P365, *p* = 0.48; P42 vs. P56, *p* = 0.99; P42 vs. P365, *p* = 0.008; P56 vs. P365, *p* = 0.03. P28, *n* = 67; P42, *n* = 119; P56, *n* = 148; P365, *n* = 60; *N* = 3 mice/group).

We examined D1R and D2R expressions in PV+ cells in the OFC and PrL (Figures 2–5). In the OFC, both the density and size of D1R puncta in PV+ cells increased at P42 and P56 when compared with those of P28 and then receded to exist at P365 (Figure 2), the density of D2R showed a similar age-related pattern, but PV+ cells at P365 still showed D2R expression (Figure 3).

In the PrL, the D1R expression in PV+ neurons was scarce across ages (Figure 4), but the D2R expression in PV+ neurons showed age-related changes similar to that of the OFC (Figure 5).

### 3.3 D1R and D2R did not co-localize in PV+ neurons in the OFC and PrL

**Figure 6:**
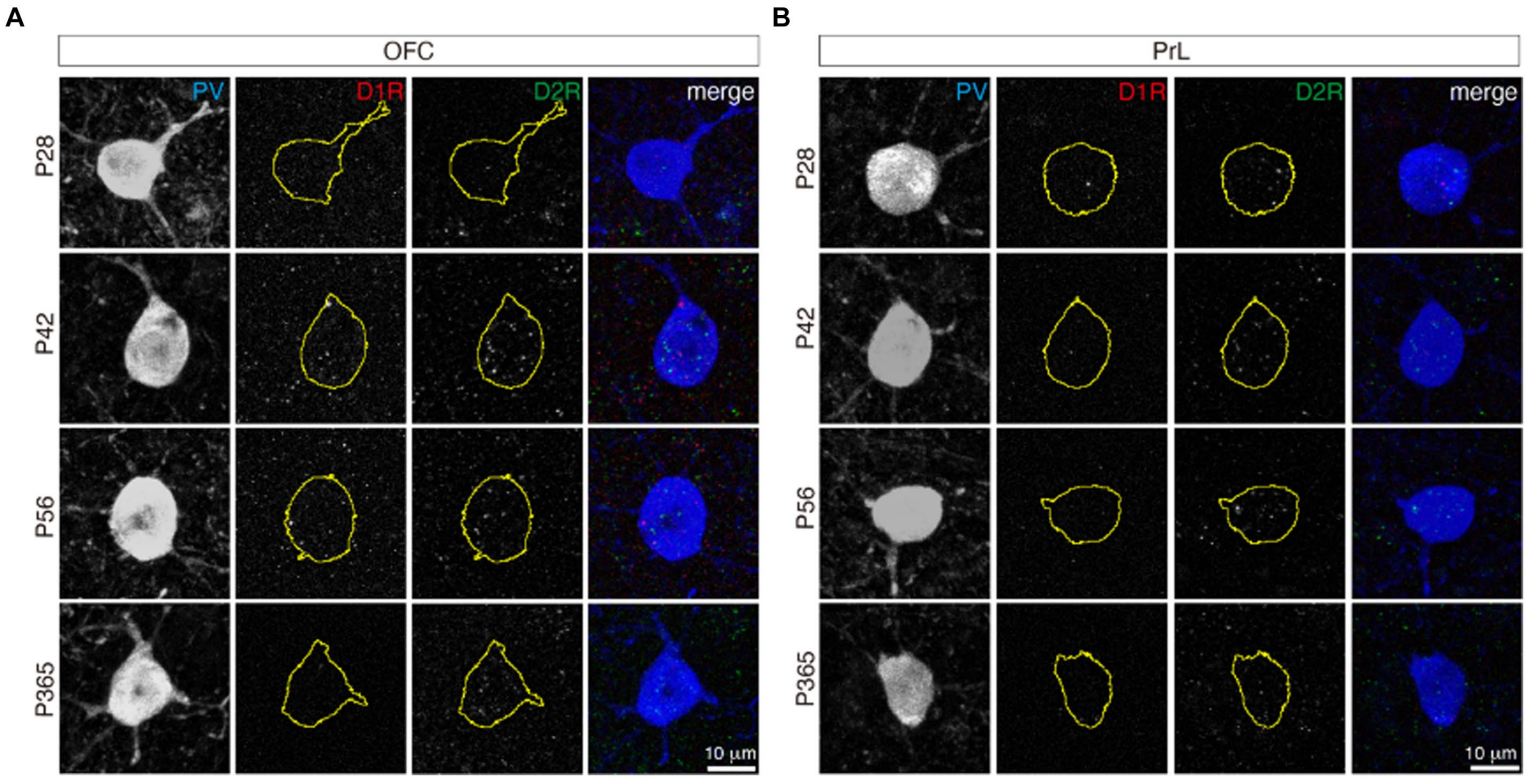
D1R and D2R did not co-localize in both the OFC and PrL. The stainings of D1R (red) and D2R (green) in PV+ neurons (blue) of the OFC **(A)** and PrL **(B)**. The yellow circles of the D1R and D2R panels indicate the related PV+ cell soma shape. Scale bars, 10 μm.

It is not clear whether PV+ neurons expressed D1/D2 receptor heteromers. D1/D2 receptor heteromers have been reported in the prefrontal cortex (Hasbi et al., 2020); nevertheless, whether D1/D2 receptor heteromers are present *in vivo* is still under debate (Frederick et al., 2015). From P28 to P365, PV+ neurons showed no co-localization of D1R and D2R in both the OFC and PrL (Figure 6), which indicates no D1/D2 receptor heteromers in those regions across age.

### 3.4 Comparisons of D1R and D2R expression in PV+ neurons of the OFC and PrL

**Figure 7:**
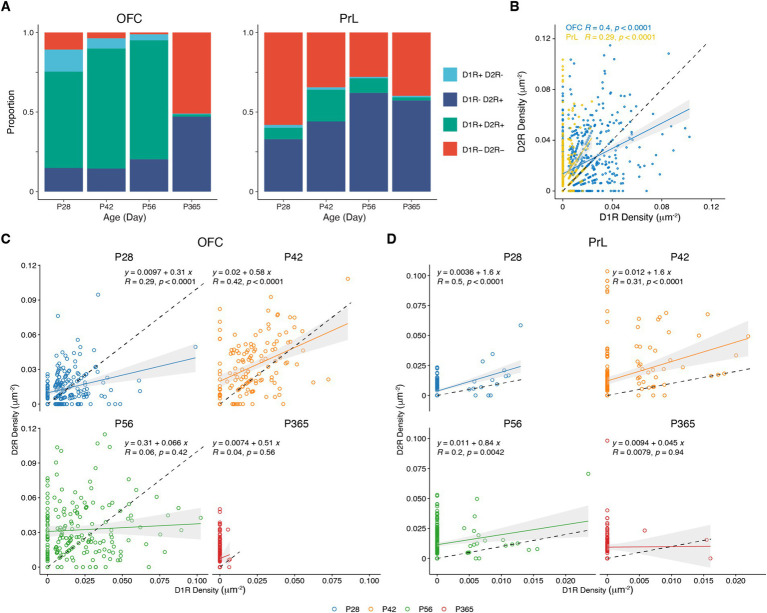
Correlation analysis of the expressions of D1R and D2R in PV+ neurons. **(A)** The proportion of D1R and D2R expressed by PV+ neurons in the OFC and PrL (*N* = 3/group). **(B–D)** Correlation analysis of density of D1R and D2R expressions in PV+ neurons in the OFC and PrL (**B**, OFC, *n* = 741; PrL, *n* = 663. **C**, P28, *n* = 215; P42, *n* = 138; P56, *n* = 182; P365, *n* = 206. **D**, P28, *n* = 167; P42, *n* = 186; P56, *n* = 208; P365, *n* = 102; *N* = 3 mice/group).

We categorized PV+ neurons in the OFC and PrL based on the expression patterns of D1R and D2R into four groups, namely D1R–D2R−, D1R + D2R−, D1R–D2R+, and D1R + D2R+ (Figure 7A and Table 1). We found that PV+ neurons in these regions showed different expression patterns of the D1R and D2R. Correlation analysis of the density of D1R and D2R in each cell of the PrL and OFC across ages further showed that overall PV+ neurons in the OFC expressed more D1R than D2R, while PV+ neurons in the PrL showed the opposite pattern (Figure 7B). Further analyses at different ages confirmed a similar region-specific pattern except for P365 when the expression D1R in PV+ neurons in both brain regions receded (Figures 7C,D).

**Table 1:** Summary of cell numbers of OFC and PrL.

Age	D1R + D2R−		D1R–D2R+		D1R + D2R+		D1R–D2R−		Total
	Cell number	Proportion	Cell number	Proportion	Cell number	Proportion	Cell number	Proportion	
**OFC**									
P28	30	0.14	32	0.15	130	0.60	23	0.11	215
P42	9	0.07	20	0.14	104	0.75	5	0.04	138
P56	9	0.05	38	0.21	133	0.73	2	0.01	182
P365	1	0.00	97	0.47	3	0.01	105	0.51	206
**PrL**									
P28	3	0.02	55	0.33	12	0.07	97	0.58	167
P42	3	0.02	82	0.44	37	0.20	64	0.34	186
P56	2	0.01	129	0.62	19	0.09	58	0.28	208
P365	1	0.01	58	0.57	2	0.02	41	0.40	102

**Figure 8:**
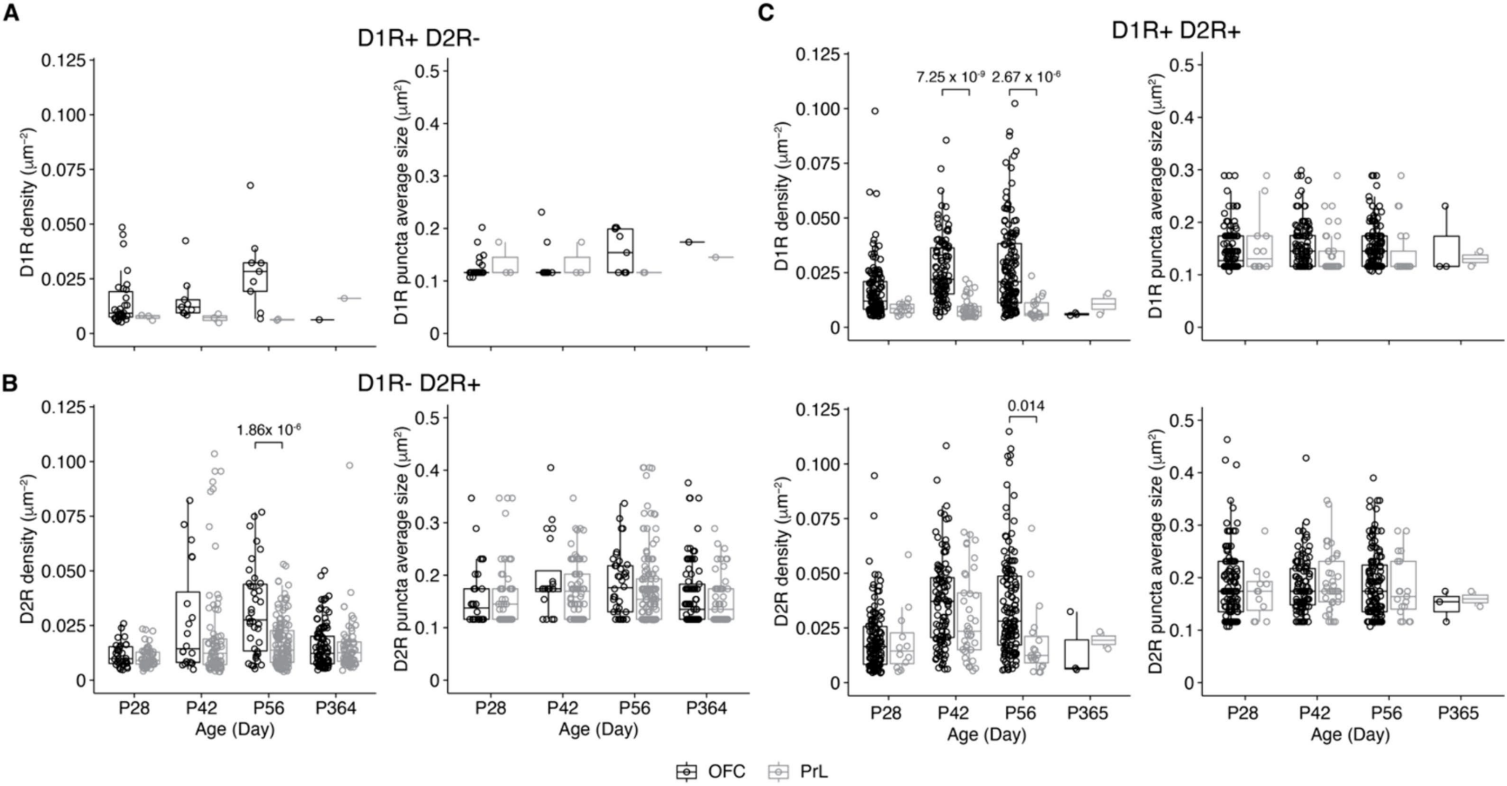
Comparisons of D1R and D2R expressions of PV+ neurons of the PrL and OFC. **(A)** Comparative analysis of the density of PV+ neurons expressing only D1R in the OFC and PrL (Left, D1R density: Two-way ANOVA, *F*_1, 50_ (region) = 0.9, *p* = 0.34, *F*_3, 50_ (Age) = 3.3, *p* = 0.03, *F*_3, 50_ (region: Age) = 1.0, *p* = 0.39; *post hoc* Tukey’s test: OFC vs. PrL, *p* = 0.04; P28 vs. P56, *p* = 0.05. Right, D1R puncta average size: Two-way ANOVA, *F*_1, 50_ (region) = 0.5, *p* = 0.50, *F*_3, 50_ (Age) = 3.6, *p* = 0.02, *F*_3, 50_ (region: Age) = 1.2, *p* = 0.31. OFC: P28, *n* = 30; P42, *n* = 9; P56, *n* = 9; P365, *n* = 1; PrL: P28, *n* = 3; P42, *n* = 3; P56, *n* = 2; P365, *n* = 1; *N* = 3 mice/group). **(B)** Comparative analysis of the density of PV+ neurons expressing only D2R in the OFC and PrL (Left, D2R density: Two-way ANOVA, *F*_1, 503_ (region) = 0.2, *p* = 0.69, *F*_3, 503_ (Age) = 16.1, *p* = 5.13 × 10^−10^, *F*_3, 503_ (region: Age) = 6.5, *p* = 2.46 × 10^−4^; *post hoc* Tukey’s test: OFC vs. PrL, *p* = 0.03; P28 vs. P42, *p* = 7.47 × 10^−7^; P28 vs. P56, *p* = 3.91 × 10^−6^; P42 vs. P365, *p* = 4.24 × 10^−4^; P56 vs. P365, *p* = 2.58 × 10^−3^; P28 of OFC vs. P42 of OFC, *p* = 5.36 × 10^−3^; P28 of OFC vs. P56 of OFC, *p* = 6.09 × 10^−7^; P28 of PrL vs. P42 of OFC, *p* = 3.49 × 10^−4^; P28 of PrL vs. P42 of PrL, *p* = 0.002; P28 of PrL vs. P56 of OFC, *p* = 6.25 × 10^−10^; P42 of OFC vs. P365 of OFC, *p* = 0.02; P42 of PrL vs. P56 of OFC, *p* = 0.003; P56 of OFC vs. P56 of PrL, *p* = 1.86 × 10^−6^; P56 of OFC vs. P365 of OFC, *p* = 3.46 × 10^−7^; P56 of OFC vs. P365 of PrL, *p* = 9.18 × 10^−6^. Right, D2R puncta average size: Two-way ANOVA, *F*_1, 503_ (region) = 0.3, *p* = 0.61, *F*_3, 503_ (Age) = 2.7, *p* = 0.05, *F*_3, 503_ (region: Age) = 0.9, *p* = 0.43; *post hoc* Tukey’s test: P56 vs. P365, *p* = 0.04. OFC: P28, *n* = 32; P42, *n* = 20; P56, *n* = 38; P365, *n* = 97; PrL: P28, *n* = 55; P42, *n* = 82; P56, *n* = 129; P365, *n* = 58; *N* = 3 mice/group). **(C)** Comparative analysis of the density of D1R and D2R co-expressed by PV+ neurons in OFC and PrL (Top-left, D1R density: Two-way ANOVA, *F*_1, 432_ (region) = 2.9, *p* = 0.09, *F*_3, 432_ (Age) = 14.8, *p* = 3.68 × 10^−9^, *F*_3, 432_ (region: Age) = 2.04, *p* = 0.11; *post hoc* Tukey’s test: OFC vs. PrL, *p* < 0.00001; P28 vs. P42, *p* = 1.44 × 10^−5^; P28 vs. P56, *p* = 3.33 × 10^−7^; P28 of OFC vs. P42 of OFC, *p* = 1.9 × 10^−5^; P28 of OFC vs. P56 of OFC, *p* = 3.48 × 10^−7^; P28 of PrL vs. P42 of PrL, *p* = 0.003; P28 of PrL vs. P56 of OFC, *p* = 0.001; P42 of OFC vs. P42 of PrL, *p* = 7.25 × 10^−8^; P42 of OFC vs. P56 of PrL, *p* = 1.08 × 10^−4^; P42 of PrL vs. P56 of OFC, *p* = 4.53 × 10^−9^; P56 of OFC vs. P56 of PrL, *p* = 2.67 × 10^−5^. Top-right, D1R puncta average size: Two-way ANOVA, *F*_1, 432_ (region) = 0.90, *p* = 0.34, *F*_3, 432_ (Age) = 1.8, *p* = 0.15, *F*_3, 432_ (region: Age) = 1.2, *p* = 0.30. Bottom-left, D2R density: Two-way ANOVA, *F*_1, 432_ (region) = 0.005, *p* = 0.95, *F*_3, 432_ (Age) = 21.4, *p* = 6.05 × 10^−13^, *F*_3, 432_ (region: Age) = 1.8, *p* = 0.15; *post hoc* Tukey’s test: OFC vs. PrL, *p* = 0.04; P28 vs. P42, *p* < 0.00001; P28 vs. P56, *p* = 1.27 × 10^−8^; P28 of OFC vs. P42 of OFC, *p* = 2.46 × 10^−10^; P28 of OFC vs. P56 of OFC, *p* = 4.71 × 10^−9^; P28 of PrL vs. P42 of OFC, *p* = 0.04; P42 of OFC vs. P56 of PrL, *p* = 0.003; P56 of OFC vs. P56 of PrL, *p* = 0.01. Bottom-right, D2R puncta average size: Two-way ANOVA, *F*_1, 432_ (region) = 0.5, *p* = 0.50, *F*_3, 432_ (Age) = 0.40, *p* = 0.76, *F*_3, 432_ (region: Age) = 0.4, *p* = 0.74. OFC: P28, *n* = 130; P42, *n* = 104; P56, *n* = 133; P365, *n* = 3; PrL: P28, *n* = 12; P42, *n* = 37; P56, *n* = 19; P365, *n* = 2; *N* = 3 mice/group).

We then compared the expression of D1R and D2R in PV+ cells of the four groups in the OFC and the PrL (Figure 8). Of the four groups, only D1R-D2R+ and D1R + D2R+ groups showed differences between the two brain regions (Figures 8B,C). Of the D1R + D2R+ group, the density of D1R in PV+ cells was higher in the OFC than that in the PrL at P42 and P56 (Figure 8C), while the density of D2R in PV+ cells was higher in the OFC than that in the PrL at P56 which is similar to the density of D2R of D1R-D2R+ group cells (Figure 8B).

Furthermore, we compared the expression of D1R and D2R in PV+ cells of the OFC and PrL between the left and right hemispheres in the brain at P56 (Supplementary Figure S1). We found no difference in both receptors in these regions between the two hemispheres, suggesting a lack of hemispheric laterality of the D1R and D2R of PV+ neurons in the OFC and PrL.

## 4 Discussion

In the present study, we showed that PV+ neurons in the OFC expressed both D1 (D1R) and D2 (D2R) receptors, while those in the PrL mainly expressed D2 receptors. D1 and D2 receptors are known for their different roles in regulating the excitability and GABAergic transmission of interneurons in the PFC. Studies suggest that D1- and D2-like dopamine receptors regulate interneuron activity and GABAergic transmissions through different mechanisms. For example, D1R but not D2R agonist enhanced spontaneous IPSCs (sIPSCs), while D2R agonist reduced miniature IPSCs (Seamans et al., 2001; Li et al., 2015). These data suggest that D1-like dopamine receptors increased the excitability of interneurons, and consistent with this finding, blockage of D1-like but not D2-like dopamine receptors abolished dopamine-induced increased excitability of fast-spiking interneurons (Gorelova et al., 2002). On the other hand, a study showed that the activation of D1-like dopamine receptors inhibited evoked-GABAergic transmission, while agonists of D2-like dopamine receptors had no effect (Gonzalez-Islas and Hablitz, 2001). Considering the different dopamine receptor expression patterns of PV+ neurons in the PrL and the OFC, dopamine might induce different functional changes of the PV+ neurons in the PRL and the OFC, which would contribute to different changes of network activity in these brain regions.

In the present study, we also observed higher proportions of PV+ neurons with D1 and D2 receptor co-expressions in the OFC than those in the PrL, and we found no co-localization of these two receptors in PV+ neurons. In the brain, D1R and D2R can form heterodimer (Perreault et al., 2014), and studies suggest the activation of such heterodimer recruits Gα_q/11_ and releases calcium from the internal stores (Rashid et al., 2007; Hasbi et al., 2009). Interestingly, dopamine increased the excitability of layer I interneurons, which was mimicked with the co-application of D1-like and D2-like dopamine receptor agonists (Wu and Hablitz, 2005), indicating a possible synergic effect of D1- and D2-like receptor interaction. However, whether PV+ neurons in the OFC and PrL also showed a similar synergic effect upon dopamine activation needs further investigation.

The PRL and OFC are two brain regions that regulate emotions, decision-making, and other cognitive processes. While they share some similarities, there are also some key differences between these two regions. The OFC is suggested as the first stage of cortical processing of the reward value-related information, with neurons that respond to the outcome and the expected value (Padoa-Schioppa and Conen, 2017; Rolls, 2019, 2021). It is worth noting that those expected-value neurons do not reflect prediction error as they keep responding to the expected reward without prediction error. Furthermore, studies have shown that OFC is involved in decision-making by representing rewards, punishments, and errors during decision-making (Rolls, 2019), while the PrL is more related to prediction error (Casado-Roman et al., 2020). The higher proportions of D1R+ PV+ neurons in the OFC might contribute to these processes.

Studies have shown that dopamine release showed age-related changes in the prefrontal cortex. For example, in young rats (2–3 months) handling stress produced an increase of dopamine higher than that of middle-aged rats (14 months), while in aged rats (30 months) stress produced no significant increases in dopamine in the prefrontal cortex (Del Arco et al., 2001, 2011). In human prefrontal cortex, D2R mRNA levels were highest in neonates, while that of D1R was highest in adolescents and young adults. Furthermore, both D1R and D2R mRNA were significantly lower in the aged cortex (Weickert et al., 2007). Age-related changes in the dopaminergic system in the PFC also contribute to the difference in cognition across ages (Hedden and Gabrieli, 2004). A human study showed age-related alterations in dopaminergic neurotransmission may contribute to under-recruitment of task-relevant brain regions during working-memory performance in old age (Backman et al., 2011).

We also observed different development changes of dopamine receptors in the PV+ neurons between the OFC and PrL. More than 80% of PV+ neurons in the OFC expressed dopamine receptors at P28, while less than 50% of those in the PrL did. At the following ages of P42 and P56, while the percentages of dopamine receptor-expressing PV+ neurons increased in both the PrL and OFC, the patterns of change differed between the two brain regions. In the OFC, the percentage of D1R+ D2R+ PV+ neurons increased with age. Meanwhile, the percentage of D1R-D2R+ PV+ neurons increased in the PrL. For mice at P365, PV+ neurons in both the PrL and OFC showed a significant reduction in the density of dopamine receptors, and the majority of those neurons only expressed D2R. Studies examined D1R and D2R expression in the cerebral cortex also found D2R expression did not change from birth to 70 weeks old while D1R expression reached a plateau around 2–3 weeks old then decreased in the prefrontal cortex in rodents (Leslie et al., 1991; Rani and Kanungo, 2006). While these studies analyzed the overall expression of those two types of dopamine receptors, the present study focused on the expression of these receptors on PV+ neurons. Considering that ~30% of inhibitory neurons in the cortex are PV+ neurons and ~ 20% of cortical neurons are inhibitory, the PV+ neurons might exhibit a different expression pattern than the overall expressions of D1R and D2R in the cortex.

In the present study, we showed the age- and region-specific expression patterns of D1 and D2 dopamine receptors in PV+ neurons of the orbitofrontal and prelimbic regions of the prefrontal cortex. Our results showed that while D1R+ PV+ neurons in both regions decreased along with aging, PV+ neurons in the OFC showed higher density and percentage of D1R and D2R expression than that of the PrL through adulthood. These results provide anatomical evidence for the understanding of PV+ neuron functions in these regions in reward-related brain functions. However, it should be noted that to fully understand the age-related changes of the dopamine system on the function of PV+ neurons, long-term monitoring of dopamine system function is necessary, such as recording of dopamine release with genetically encoded fluorescent biosensors, *in vivo* electrophysiological recording of neuronal activity, or PET imaging for dopamine receptor availability. Furthermore, we did not include female mice in the present study, due to considerations of that female mice show variation in dopamine function and dopamine receptors due to sexual hormones related to the menstrual cycle. Studies have shown that both steroid hormones 17ß-estradiol (E2) and progesterone (PROG) are endogenous modulators of dopaminergic transmission in female rodents (Diekhof and Ratnayake, 2016). Combined with the relative low density of D1R and D2R in PV+ neurons, it potentially requires females with the same menstrual cycle period for accurate comparisons between ages. Furthermore, as suggested in a recent review, rodents also experience oopause for roughly 50% of their life (Winkler and Goncalves, 2023), and menopause-related deficiency in estrogen attenuated dopamine activity (Gorzek et al., 2007). Another caveat in the present study is that we did not study the possible subregional difference in both the OFC and PrL. Recent studies have shown that the difference of subregional targeting of the dorsal striatum and other brain regions from the PFC (Choi et al., 2023; Tan et al., 2023; Tudi et al., 2024); however, we did not address this problem since the lack of anatomical or biochemical criteria for the subregions made the comparisons across age groups difficult.

## Data availability statement

The original contributions presented in the study are included in the article/Supplementary material, further inquiries can be directed to the corresponding author.

## Ethics statement

The animal study was approved by Peking University Animal Care and Use Committee. The study was conducted in accordance with the local legislation and institutional requirements.

## Author contributions

JD: Data curation, Formal analysis, Investigation, Methodology, Project administration, Software, Writing – review & editing. XW: Investigation, Methodology, Writing – review & editing. ZH: Investigation, Methodology, Writing – review & editing. JT: Investigation, Methodology, Writing – review & editing. WZ: Conceptualization, Formal analysis, Funding acquisition, Software, Supervision, Writing – original draft, Writing - review & editing.
