# Supplementary material for: Age-related changes of dopamine D1 and D2 receptors expression in parvalbumin-positive cells of the orbitofrontal and prelimbic cortices of mice
Source: Front Neurosci. 2024 Jun 6;18:1364067. doi: 10.3389/fnins.2024.1364067 (PMC11187244; doi:10.3389/fnins.2024.1364067)
Supplement: Supplementary file 1 [file Table_1.DOCX]

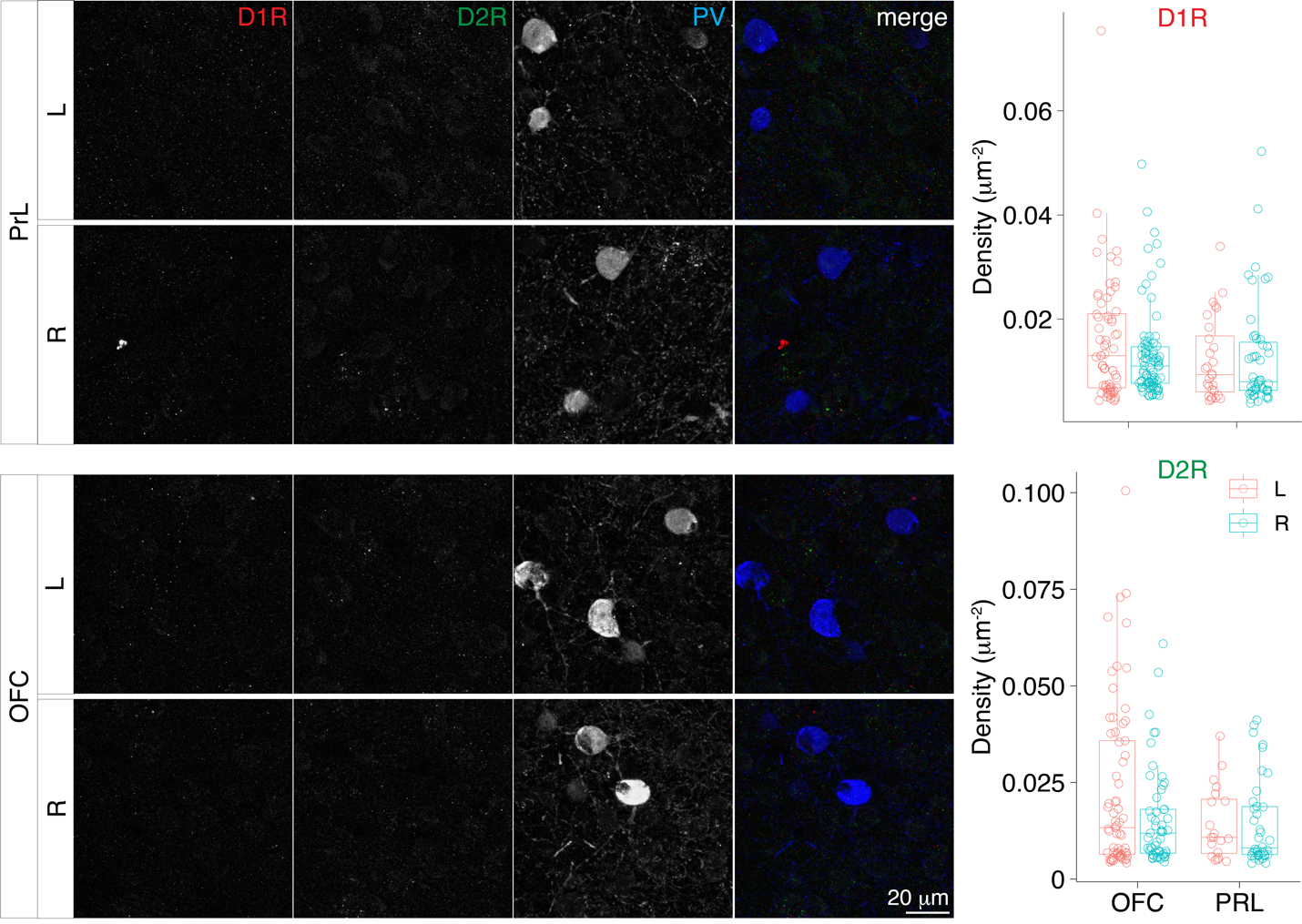
Supplementary Figure 1

SFigure1. D1R and D2R did not show lateralization in both the OFC and PrL at P56. D1R: left PrL, n= 28; right PrL, n= 47; left OFC, n= 64; right OFC, n= 82. D2R: left PrL n= 20; right PrL, n= 38; left OFC, n= 65; right OFC, n= 53. N= 2mice.
